# Restructuring Physical Therapy Education After COVID-19: A Narrative Review on the Global Perspectives and the Emerging Role of Hybrid Learning Models

**DOI:** 10.7759/cureus.88034

**Published:** 2025-07-15

**Authors:** Kazuto Kikuchi

**Affiliations:** 1 Physical Therapy, Akita Rehabilitation College, Akita, JPN

**Keywords:** clinical skills acquisition, digital transformation in physical therapy education, educational equity and access, hybrid education, student motivation and engagement

## Abstract

The COVID-19 pandemic rapidly transformed physical therapy (PT) education from traditional face-to-face instruction to online and hybrid models worldwide. While online education effectively supports theoretical knowledge acquisition, it falls short in developing hands-on clinical skills, highlighting the necessity of integrating in-person training. Various countries reported benefits and challenges of online learning, including issues with learning environments, faculty ICT skills, and student motivation. Hybrid education models combining online lectures with practical face-to-face sessions emerged as optimal solutions. Future PT education requires flexible, sustainable, and learner-centered approaches grounded in educational technology and human-centered design. Key priorities include standardizing hybrid models, enhancing faculty support, reforming assessment methods, and ensuring equitable access to digital resources. Overall, PT education faces a pivotal opportunity to evolve into a resilient system balancing educational quality with accessibility and adaptability, guided by comprehensive, evidence-based strategies.

## Introduction and background

The COVID-19 pandemic, which began in 2020, forced higher education institutions across the globe to abruptly transition from traditional, in-person teaching to remote, online formats. In physical therapy (PT) education, this shift had significant consequences. Many students were unable to complete required clinical hours, delaying their academic progression and graduation, and ultimately impacting the availability of qualified healthcare professionals [1].

A defining feature of PT education is the emphasis on hands-on training, particularly through preceptorships and clinical clerkships-structured, supervised experiences that take place in clinical settings. These components are essential for developing practical competencies, including manual techniques, patient communication, and clinical decision-making. The sudden pivot to online delivery disrupted these experiences and prompted a reexamination of how PT education could maintain quality and competency-based standards in a digital environment.

In response, many programs adopted hybrid education models, combining online instruction for theoretical content with in-person sessions for practical skill development. While hybrid formats were already being explored before the pandemic, COVID-19 catalyzed their widespread implementation across global health professions education.

International responses varied. In Australia, PT programs initially moved fully online, but student dissatisfaction with remote skill training led to a shift toward hybrid formats [2]. In the United Kingdom, students acknowledged the flexibility of online education but reported declines in comprehension, motivation, and satisfaction, especially with practical components, affecting 79% of respondents [3]. By contrast, Italian PT programs observed improved academic outcomes and satisfaction comparable to traditional instruction [4]. At Harvard Medical School, a fully online radiology clerkship demonstrated notable educational efficacy despite its remote format [5]. However, many institutions struggled with faculty preparedness for digital teaching, leading to inconsistency in educational delivery [3].

Despite rapid adaptations and growing experience with digital learning tools, there remains a limited synthesis of global evidence regarding implementation strategies, educational outcomes, and best practices for hybrid PT education. Most existing reports are country- or institution-specific, limiting generalizability.

This narrative review aims to bridge that gap by synthesizing peer-reviewed literature published between 2019 and 2025, focusing on digital and hybrid educational strategies in PT and related healthcare fields. Specifically, this review compares the strengths and limitations of online and in-person instruction, evaluates learning outcomes and student satisfaction, and highlights the emerging role of hybrid models in maintaining educational continuity. By identifying key patterns and unresolved challenges, this work seeks to inform future strategies for building flexible, effective, and resilient PT education systems in a post-pandemic world.

## Review

Methods: literature search strategy

Study Design

This narrative review was conducted in partial alignment with the PRISMA (Preferred Reporting Items for Systematic Reviews and Meta-Analyses) 2020 guidelines, as applicable to narrative syntheses. The primary objective was to synthesize peer-reviewed literature describing digital education strategies, including online, hybrid, remote clinical, and simulation-based learning, implemented in PT and related healthcare professional education during the COVID-19 pandemic. A narrative approach was chosen to accommodate the heterogeneity in study designs and educational interventions across countries and disciplines.

Search Strategy

A comprehensive search was carried out using the PubMed database to identify articles published between January 1, 2019, and June 30, 2025. The search employed both Medical Subject Headings (MeSH) and free-text terms to maximize coverage. The complete search strategy is provided in Table 1. No language restrictions were applied, and Japanese-language publications indexed in PubMed were included.

**Table 1:** Overview of the literature search strategy

Item	Details
Databases	PubMed, Google Scholar, Ichushi Web (Japan Medical Abstracts Society), CiNii Articles
Search Period	January 1, 2019 – June 30, 2025
Target Literature	Peer-reviewed original articles (regardless of language: English and Japanese included)
Target Fields	Physical therapy education, health professions education (e.g., medicine, nursing, rehabilitation)
Educational Methods	Online learning, hybrid education, distance education, simulation-based education, virtual reality (VR), digital clinical placements, telehealth
Search Strategy (PubMed)	("physical therapy"[Title/Abstract] OR physiotherapy[Title/Abstract] OR "physical therapy modalities"[MeSH] OR "health professions education"[MeSH] OR "medical education"[MeSH] OR "nursing education"[MeSH]) AND("distance learning"[MeSH] OR e-learning[Title/Abstract] OR "online education"[Title/Abstract] OR "remote learning"[Title/Abstract] OR "digital learning"[Title/Abstract] OR "hybrid learning"[Title/Abstract] OR simulation[Title/Abstract] OR telehealth[Title/Abstract] OR "virtual reality"[Title/Abstract]) AND("COVID-19"[MeSH] OR COVID-19[Title/Abstract] OR coronavirus[Title/Abstract] OR SARS-CoV-2[Title/Abstract]) Supplementary terms used in additional manual searches included: “COVID-19,” “distance education,” “physical therapy education,” “preceptorship,” and “clinical clerkship.”
Language Restriction	None (Japanese-language articles included; manually supplemented if not indexed in PubMed)
Supplementary Search	Additional manual searches in Google Scholar, Ichushi Web, and CiNii Articles for Japanese-language literature not indexed in PubMed, using key terms such as “COVID-19,” “physical therapy education,” and “distance education”
Literature Selection	Screening based on title and abstract, followed by full-text review for final inclusion

To capture additional relevant studies published in Japan, a manual search was conducted using Google Scholar, Ichushi Web (Japan Medical Abstracts Society), and CiNii Articles. These platforms enabled the retrieval of gray literature, academic bulletins, and domestic journals not indexed in PubMed. Supplementary search terms included “COVID-19”, “distance education”, “physical therapy education”, “preceptorship”, and “clinical clerkship”. Reference lists of included studies were also screened to identify any additional relevant publications.

As a result, a total of 140 records were identified: 113 through PubMed and 27 through manual searches. After removing 21 duplicates, 119 records were retained for screening.

Inclusion and Exclusion Criteria

Studies were considered eligible for inclusion if they met the following criteria: they were original peer-reviewed articles; they focused on digital education approaches such as online, hybrid, remote clinical, or simulation-based instruction; they involved PT or related healthcare professional education, including nursing and medical education; and they were conducted during or directly relevant to the COVID-19 pandemic. Articles published between 2019 and 2025 were eligible.

Studies were excluded if they were conference abstracts, editorials, commentaries, or opinion pieces lacking empirical data; if they focused solely on technological infrastructure without evaluating educational outcomes; or if they were not available in full text.

Screening Process

**Figure 1:**
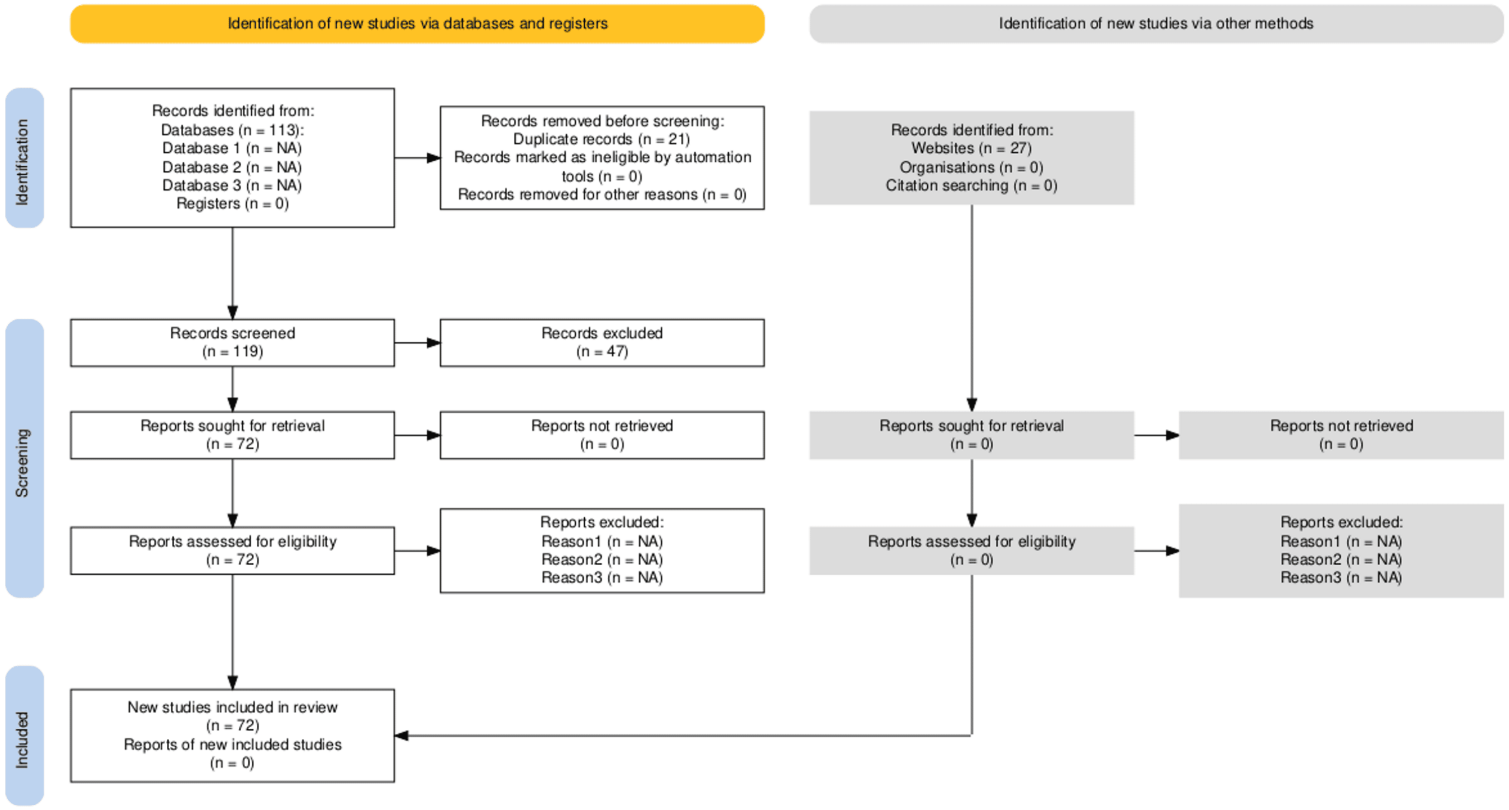
PRISMA 2020 flow diagram of literature identification, screening, and inclusion in this narrative review This flow diagram illustrates the literature identification and screening process for this narrative review. A total of 140 records were initially identified—113 through PubMed and 27 through manual searches using Google Scholar, Ichushi Web, and CiNii Articles. The PubMed search strategy combined MeSH terms and free-text keywords related to physical therapy education, digital learning, and COVID-19. Supplementary search terms such as “COVID-19”, “distance education”, “physical therapy education”, “preceptorship”, and “clinical clerkship” were used for manual searches. No language restrictions were applied, and the search covered publications from January 1, 2019, to June 30, 2025. After removing 21 duplicates, 119 records remained for screening. Following title and abstract review, 47 records were excluded. The remaining 72 full-text articles were assessed for eligibility, and all were included in the final synthesis.

Given the narrative nature of this review, the screening process was conducted by a single reviewer (the author). Titles and abstracts of the 119 unique records were first screened against the inclusion and exclusion criteria, resulting in the exclusion of 46 records. The remaining 72 articles underwent full-text review, all of which met the eligibility criteria and were included in the final synthesis. The study selection process is summarized in a PRISMA-style flow diagram (Figure 1).

Thirteen of the included articles were published in Japanese, ensuring comprehensive coverage of both domestic and international literature on digital and hybrid education in PT and related healthcare fields.

Quality Assessment

Although this review did not include a formal risk-of-bias assessment using standardized tools, an informal appraisal of methodological quality was conducted. Each study was evaluated for the clarity of its research question, the appropriateness of its methodology, the transparency of outcome reporting, and its relevance to the theme of digital and hybrid education. Studies lacking sufficient methodological rigor or educational relevance were excluded during the screening process.

Review of online education in medical and rehabilitation sciences

Global Implementation and Modality Shifts

The COVID-19 pandemic disrupted clinical training across healthcare education, leading to the suspension or postponement of preceptorships and clinical clerkships in many countries. In response, institutions rapidly transitioned to virtual case-based learning, simulation-based modules, and group activities conducted via online platforms such as Zoom [5]. A scoping review reported that both synchronous (real-time) and asynchronous (on-demand) digital learning modalities had a positive impact on PT students’ knowledge acquisition, overall learning experience, and satisfaction [6]. Notably, interactive tools such as gamification and virtual reality (VR) technologies showed high educational value. However, the shift to online learning also presented challenges, including reduced self-efficacy and limited face-to-face interaction. In nursing education, high-fidelity simulation was found to be a valid substitute for up to 50% of traditional clinical training, with no significant differences observed in student competency or national exam pass rates [7].

Country-Specific Implementation: The Case of Japan

In Japan, formal online learning policies were introduced in 2020 following guidance from the Ministry of Education, Culture, Sports, Science and Technology (MEXT). Digital platforms such as Google Classroom, Zoom, and Moodle became standard tools for delivering lectures and managing coursework. To compensate for the loss of clinical training, some programs conducted on-campus practical sessions under controlled conditions. Student satisfaction with these arrangements varied across academic years, with younger students often requiring more structured support [8]. Reported challenges included inadequate home learning environments and physical strain during extended screen time, emphasizing the need for instructional design that addresses ergonomic and motivational factors [9].

Innovative approaches such as tele-supervised clinical placements were piloted at several institutions. These incorporated individualized mentoring and peer feedback to maintain instructional quality [10]. Furthermore, hybrid teaching models that combined real-time engagement tools like Kahoot! with asynchronous video lectures were met with high satisfaction, though students and faculty alike reported increased workload and complexity [11]. Disparities in faculty information and communication technology (ICT) proficiency and unequal access to high-speed internet among students also highlighted the issue of digital inequality [12]. As a strategic response, some universities began implementing Business Continuity Management (BCM) frameworks to ensure educational resilience and preparedness for future disruptions [13].

Comparative Effectiveness of Online and Hybrid Education

While online education has demonstrated effectiveness in delivering theoretical content and enhancing access, it remains limited in supporting the acquisition of hands-on clinical skills and interpersonal competencies. In Australia, PT programs initially transitioned entirely to e-learning formats, but growing concerns about practical skill development led to the adoption of hybrid models that integrated in-person skill labs with online lectures [2,6]. Italian studies revealed improved academic performance in online environments, with student satisfaction levels comparable to traditional classroom settings [4]. In the United Kingdom, hybrid learning models were well received and deemed particularly effective for balancing flexibility with essential clinical training [14,15].

Collectively, these international experiences suggest that hybrid education-defined as the deliberate integration of digital and face-to-face components-offers a viable and sustainable pathway for the future of healthcare professional education. By combining the scalability of online delivery with the experiential value of in-person instruction, hybrid models are positioned to address both educational quality and learner accessibility in the evolving post-pandemic landscape.

Benefits and challenges of online education in PT education

Advantages of Online Learning

The transition to online education during the COVID-19 pandemic enabled PT programs to overcome traditional constraints of time and location. This shift contributed to enhanced student engagement and learning motivation [16]. A study conducted in Morocco emphasized that the quality of the learning environment, particularly in terms of organization and clarity, was closely associated with increased motivation [17]. Moreover, even in remote or asynchronous settings, students reported improvement in skill acquisition and overall satisfaction, especially when feedback was provided iteratively and promptly [18].

Online learning has also expanded access to educational opportunities, particularly in low- and middle-income countries [19]. In these contexts, blended learning approaches that integrate digital content with occasional in-person instruction have become increasingly common. The use of multimedia and interactive content has been associated with improved academic outcomes in both medical and rehabilitation education [20]. In postgraduate programs, digitally mediated mentoring, or “e-mentoring,” has been shown to support the development of practical skills and enhance learners’ self-efficacy [21].

Limitations in Acquiring Practical and Interpersonal Skills

Despite its strengths in delivering theoretical knowledge, online education presents notable limitations when applied to the development of hands-on clinical competencies. A large-scale review concluded that although e-learning supports the acquisition of factual and conceptual knowledge, it frequently lacks opportunities for direct clinical exposure [22,23]. In regions with poor internet connectivity or limited access to equipment, these limitations are further exacerbated [24].

Reports from nursing education highlight the psychosocial impact of online instruction, with students experiencing feelings of isolation and difficulty engaging in the development of psychomotor and interpersonal communication skills. Studies conducted in Jordan, the United Kingdom, and Ireland have similarly identified environmental constraints, such as inadequate access to course materials and inconsistent digital infrastructure, as barriers to successful learning outcomes [25,26]. Although virtual clinical training alternatives have been piloted with varying degrees of success, they are generally not perceived as fully equivalent to traditional in-person preceptorships or clerkships [27,28].

Maintaining Motivation and Focus in Online Environments

The widespread and sudden shift to digital formats had a marked effect on student mental health and academic focus. Reduced opportunities for peer interaction and a lack of structured learning environments contributed to lower levels of concentration and emotional well-being [29]. Instructors responded by incorporating interactive features such as real-time polling and live-streamed lectures to enhance engagement. However, challenges such as time zone differences, domestic distractions, and weakened teacher-student rapport continued to hinder learning.

In addition to the challenges faced by students, educators were also burdened by the need to design, develop, and deliver content suitable for online platforms. A SWOT (Strengths, Weaknesses, Opportunities, Threats) analysis of pandemic-era education strategies highlighted the importance of pedagogical responsiveness and flexibility in maintaining student engagement and learning outcomes in virtual environments [30].

Faculty Burden and ICT-Related Disparities

The digital transformation of PT education also brought new responsibilities for faculty. Many were required to rapidly develop ICT (Information and Communication Technology) skills, redesign their curricula for online delivery, and provide individualized support to students. These demands created considerable psychological stress and increased workloads for educators [31,32].

Simultaneously, disparities in student access to digital tools and stable internet connections emerged as a significant source of educational inequity. The mental health of teaching staff became an area of growing concern, prompting institutions to reevaluate their support structures [33]. Learning management systems (LMSs), such as Moodle, proved useful for promoting self-directed and collaborative learning. However, variations in faculty competence with these systems and with online instructional design continue to present implementation challenges. Nevertheless, LMS platforms are expected to remain a central component of healthcare education's digital future, offering scalable and structured approaches to content delivery and learner engagement [34].

Educational effectiveness of online and in-person instruction: international evidence

Academic Performance, Satisfaction, and Motivation

The global adoption of online learning during the COVID-19 pandemic catalyzed a series of comparative studies evaluating its effectiveness relative to traditional in-person instruction. In the field of cardiopulmonary PT, newly graduated physical therapists who experienced both modalities reported diminished self-efficacy in practical skill domains. This suggests that the modality of instruction-particularly the absence of in-person guidance-may adversely affect students' clinical confidence and perceived readiness for professional practice [35].

In anatomical education, digital tools such as three-dimensional visualization software were generally well received by students. Nevertheless, face-to-face instruction yielded significantly higher academic performance in formal assessments, indicating that digital engagement alone may not be sufficient for deep comprehension [36]. In Spain, modifications to student study habits during the lockdown period contributed to improved academic outcomes, suggesting that contextual factors also influence learning effectiveness [37]. Similarly, a medical school in Dubai reported that while the rapid transition to online teaching ensured instructional continuity, it fell short in developing hands-on competencies [38].

Despite improvements in access to education and gains in digital literacy, several studies have reported negative impacts on student engagement. Reduced concentration, limited opportunities for interaction, and insufficient clinical exposure were common concerns [39]. A survey conducted at a French medical school found high satisfaction with the digital learning environment, yet students expressed a marked preference for small-group, in-person sessions. This preference reflects the continued value of interpersonal engagement in professional training [40].

In pediatric PT education, student self-efficacy improved across face-to-face, online, and hybrid instructional formats. However, clinical exposure appeared particularly influential in enhancing communication-related competencies, which are critical for patient interaction [41]. A study analyzing students at varying academic stages noted that although online formats were perceived as convenient and informative, many participants rated the overall quality of digital instruction as inferior to that of conventional classroom settings [42].

Moreover, studies on small-group seminars revealed that learning outcomes were relatively consistent across instructional modes. However, students consistently rated face-to-face sessions higher in terms of fostering interpersonal relationships and communication, while online seminars were valued for their logistical efficiency and accessibility [43].

Development of Practical and Clinical Decision-Making Skills

The COVID-19 pandemic significantly disrupted clinical training opportunities across health professions education, compelling institutions to develop innovative alternatives. One such example is the virtual placement model implemented by Connect Health, which integrated telehealth consultations with structured online exercise classes. This model allowed for a scalable and timely response to clinical placement shortages; however, limitations in hands-on skill development remained a concern [1].

For technical competencies such as skinfold measurement and blood pressure monitoring during exercise, studies indicated that students enrolled in fully online programs demonstrated lower performance outcomes compared to those participating in face-to-face or hybrid instructional models [44]. By contrast, blended learning-combining online theoretical instruction with on-campus practical sessions-was associated with stronger collaborative learning and higher academic achievement. A study conducted in India reported that students perceived blended formats more favorably than fully digital ones, particularly in areas involving teamwork and applied clinical reasoning [45].

Ensuring educational quality in PT programs during and beyond crisis periods requires the intentional design of hybrid learning environments. These environments must be grounded in established instructional theory and supported by appropriate technological infrastructure. In addition, faculty development is essential for the successful integration of these methods into curricula. As the educational landscape continues to evolve, the development of a flexible, evidence-based, and technologically supported curriculum will be vital for preparing students to meet the demands of contemporary clinical practice [46].

Hybrid education in healthcare: potential and challenges

Educational Impact of Blended Learning Models

In the post-pandemic landscape, hybrid education-defined as the strategic integration of online and face-to-face instruction-has become increasingly prevalent in health professions education. Numerous studies have evaluated its effectiveness across various contexts.

At a South African medical university, third-year students participated in Objective Structured Clinical Examinations (OSCEs) across three formats: fully online, fully in-person, and hybrid. The blended group showed the highest overall performance, especially in psychomotor domains, with an average score nearing 90% [47]. This suggests that hybrid models may support both theoretical understanding and skill acquisition.

In the United States, hybrid Doctor of Physical Therapy (DPT) programs have expanded to 21 states, demonstrating promising educational and societal outcomes. These programs have not only increased enrollment and racial diversity but also maintained high graduation (96-99%) and employment rates [48]. One specific curriculum reported student satisfaction rates between 97-98%, with a licensure exam pass rate of 97%. This model successfully integrated asynchronous and synchronous online learning with intensive in-person laboratory training [14].

Other studies support the value of preparatory digital materials. For instance, a 16-week hybrid anatomy course found that online pre-laboratory quizzes were strong predictors of academic success, reinforcing the role of structured pre-class engagement [49]. The use of narrated instructional videos and checklists enhanced the quality of hands-on training while reducing faculty workload and promoting active, student-centered learning [50].

A scoping review by Pagels et al. revealed that nearly half of the digital tools adopted in PT education since 2010 have been implemented within hybrid models [6]. Among these tools, gamification and VR technologies were particularly effective in boosting learner motivation. However, challenges persisted, such as reduced self-efficacy and limited interpersonal interaction, underscoring the need for carefully balanced instructional design.

Support for hybrid education has also grown among clinical instructors. In one survey, 96.3% rated students trained under hybrid DPT programs as well-prepared for clinical practice. In addition, 89% of respondents considered the hybrid format equivalent or superior to traditional instruction. Although initial skepticism was common, direct clinical experience often shifted perspectives toward a more favorable evaluation [51].

Continuing medical education (CME) has similarly benefited from hybrid approaches. A study from Saudi Arabia reported high satisfaction with online CME, particularly due to its flexibility and accessibility. However, persistent limitations in developing practical skills highlighted the continued importance of in-person instruction for applied learning domains [52].

Balancing Flexibility With Educational Quality

While hybrid education offers greater flexibility in learning time and location, maintaining educational quality and student motivation remains an ongoing challenge. A study from Germany compared cohorts receiving face-to-face education in 2019 and online instruction in 2020. The results showed a slight decline in motivation across both groups, with no statistically significant difference. Interestingly, online students benefited from self-assessment tools and written assignments, while in-person learners demonstrated higher engagement through classroom attendance [53].

These findings suggest that motivation and engagement can be preserved in online environments when course design emphasizes structured feedback, interactivity, and clear learning objectives. However, the absence of spontaneous interaction in virtual settings may limit opportunities for informal learning and peer support.

Role of Video-Based Learning in PT Education

Video-based learning (VBL) has emerged as a valuable tool in PT education, offering flexibility and the opportunity for repeated viewing of complex procedures. One study evaluating the use of VBL in teaching proprioceptive neuromuscular facilitation (PNF) techniques - a method for improving muscle function-found no significant differences in students' practical performance when compared to face-to-face instruction. However, the VBL group outperformed their peers on knowledge assessments and reported high satisfaction with the self-paced, accessible format [54].

Despite its benefits, VBL is not without limitations. Students reported difficulties in self-assessment, comprehension of complex material without live demonstration, and managing their time effectively. These challenges highlight the importance of incorporating timely instructor feedback and instructional scaffolding, such as guided checklists and interactive elements, to support learning outcomes in hybrid and digital settings.

CME in rehabilitation professions: diversification and future perspectives in educational assessment

Diversification of CME Strategies

The COVID-19 pandemic accelerated the diversification of CME for rehabilitation professionals, particularly through the adoption of digital and hybrid instructional models. This diversification has been accompanied by innovations in the assessment of educational outcomes.

One notable development is the virtual Objective Structured Clinical Examination (vOSCE), which has emerged as a reliable tool for remote competency assessment. In a multi-site implementation across three residency programs, 94% of participants found the vOSCE useful, with no significant difference in performance compared to traditional in-person assessments. It was especially effective in evaluating communication skills, indicating its potential for broader application in remote clinical assessment environments [55].

Simulation-based education (SBE) has also gained traction. In pediatric PT education, SBE significantly enhanced students’ self-efficacy, particularly in domains related to clinical decision-making. Approximately 80% of participants expressed high levels of satisfaction, emphasizing the value of experiential, scenario-based training in developing core clinical competencies [56].

Tele-rehabilitation education has followed a similar trajectory. For example, a South African university implemented a stepwise integration model using tools such as e-portfolios, self-recorded clinical videos, and structured peer feedback. Although its direct impact on practical examination scores was limited, students demonstrated increased study time and improved reflective thinking-key elements for lifelong learning and clinical reasoning [57].

Integration of Technology-Enhanced Education

The effectiveness of technology-enhanced learning strategies in CME has been supported by a growing body of evidence. A meta-analysis demonstrated that formats such as flipped classrooms and interactive digital applications significantly improved both knowledge acquisition and practical performance, at times outperforming conventional face-to-face instruction [58]. In Pakistan, a study integrating video-based learning and simulation in a flipped-classroom model resulted in improved OSCE scores, enhanced self-efficacy, and increased student satisfaction. Although implemented in a resource-limited setting, the program was aligned with sound educational theory, suggesting its replicability, albeit with the need for further long-term evaluation [59].

VR, virtual simulation, and hybrid learning models have rapidly become mainstream in post-pandemic CME. A systematic review of 27 studies reported that VR-integrated training yielded skill acquisition outcomes equal to or superior to those of face-to-face training (mean difference = 1.93; 95% CI: 1.22-2.64). Learner satisfaction was consistently high across studies [60].

A separate scoping review focused specifically on VR-based education and confirmed improvements in technical skills, empathy, and mental well-being. However, several limitations were noted, including variability in evaluation methods, technological standards, and overall evidence quality. These findings underline the urgent need for standardized assessment frameworks and user experience (UX) evaluations to ensure consistency across platforms and contexts [61].

Hybrid virtual simulations (HVS), such as those combining video conferencing tools like Zoom with standardized patients, were positively evaluated by both learners and educators. These simulations were particularly effective in teaching communication skills and interprofessional collaboration. Despite favorable subjective outcomes, the lack of objective metrics limits the comprehensive evaluation of their educational impact [62].

Systematic reviews of VR-based educational tools support their use in procedural training and remote instruction. However, significant gaps remain in our understanding of their cost-effectiveness, long-term impact, and scalability across diverse healthcare settings [63].

Challenges and future directions in educational assessment

Innovative approaches to clinical skills training have also been explored. A virtual placement in chronic pain management demonstrated that students were able to meet learning objectives and improve clinical competencies in a fully digital environment. This example illustrates the feasibility of using telehealth-based training for practical education. However, further refinement in digital infrastructure and comparative research with traditional placement models will be essential for broader adoption [64].

A pilot randomized controlled trial comparing VR to conventional training for nasopharyngeal swab techniques found that the VR group achieved higher skill scores and maintained performance gains one month later. Despite these promising results, the small sample size and limited design scope highlight the need for replication in larger, multicenter trials [65]. Similarly, asynchronous VR case scenarios have been associated with enhanced practical skills and learner satisfaction. However, the absence of real-time feedback and objective performance metrics was identified as a major limitation, reinforcing the need for more robust evaluation systems and cost-effectiveness analysis [66].

Hybrid medical simulation (HMS), which integrates human actors with wearable devices to simulate realistic clinical scenarios, has shown potential in enhancing interpersonal, technical, and decision-making skills. Compared to high-fidelity mannequins, HMS offers a more cost-effective and immersive learning experience. Nonetheless, challenges remain in standardizing actor responses and developing scalable, validated assessment frameworks [67].

In summary, the landscape of CME in rehabilitation professions is undergoing a significant transformation through the incorporation of digital technologies such as VR, simulation, and hybrid learning models. These innovations hold promise for enhancing learner engagement, improving skills acquisition, and increasing access to flexible education. However, their success depends on the development of standardized implementation protocols, robust outcome evaluations, and long-term studies to ensure educational efficacy and sustainability.

Future directions and recommendations: optimizing hybrid education and envisioning the future of digital learning

The emergence of hybrid and digitally enhanced learning models during the COVID-19 pandemic has significantly contributed to the continuity and adaptability of health professions education. As these models become more embedded in educational practice, future developments must prioritize instructional strategies that are evidence-based, technologically supported, and sustainably implemented.

A recent study investigating learning approaches among PT students revealed that 97.6% employed a deep learning strategy focused on constructing meaning and conceptual understanding. In contrast, surface learning (2.0%) and strategic approaches (0.4%) were minimally utilized, indicating a limited dependence on rote memorization or test-oriented tactics. Although no significant differences were observed by gender or institution, the study highlighted that students' support needs varied across academic stages-particularly between basic science and clinical education-underscoring the necessity for adaptable and learner-centered instructional design tailored to developmental levels [68].

Innovative instructional models, such as triple hybrid simulations that integrate in-person, online, and virtual patient-based components, have shown promising results in enhancing clinical reasoning, understanding of telehealth environments, learning motivation, and self-efficacy. However, these models also pose challenges, including technical limitations and increased faculty workload for preparation. These findings suggest a need for further empirical validation through randomized controlled trials and collaborative, multi-institutional research initiatives that can establish generalizable evidence [69].

Immersive VR, particularly when delivered through head-mounted displays (HMDs), has shown promise in supporting procedural learning and emergency care training that transcends time and spatial constraints. However, its broader application faces challenges such as simulator sickness, cognitive overload, high development and implementation costs, and a lack of standardized assessment instruments. Most existing studies have been limited to small sample sizes and single-center designs, making multicenter collaboration and the development of validated, context-sensitive evaluation scales a high priority [70].

Virtual simulated placements (VSPs) have also gained traction as alternative strategies for clinical training. A scoping review of 28 studies indicated that VSPs effectively promoted clinical reasoning and learner engagement. Despite these benefits, the widespread adoption of VSPs has been limited by concerns regarding long-term educational outcomes, interprofessional collaboration, cultural adaptability, and variability in evaluation metrics. Additionally, technological infrastructure and financial sustainability remain barriers to implementation. To facilitate broader adoption, there is a pressing need to develop internationally applicable frameworks with standardized learning objectives and measurable educational outcomes [71].

Looking ahead, the optimization of hybrid and digital education in PT and related healthcare disciplines will require a coordinated strategy encompassing several key elements. These include designing instruction that is responsive to learner diversity and educational stage, implementing standardized and empirically tested models that ensure quality and consistency, and establishing sustainable technological and financial systems that support scalability and access. Furthermore, interdisciplinary research that integrates educational theory, digital innovation, and clinical practice will be essential for bridging the gap between conceptual models and real-world implementation.

Striking a balance between flexibility and educational quality will remain a central challenge. Therefore, collaboration among educators, healthcare professionals, institutional leaders, and policymakers is crucial. By aligning pedagogical innovation with practical feasibility, the long-term success of digitally enhanced education in the health professions can be secured.

## Conclusions

This narrative review examined the transformation of PT and related healthcare professional education in the wake of the COVID-19 pandemic. It focused on the widespread adoption of online learning, its comparison with traditional in-person instruction, and the educational outcomes associated with each modality. The pandemic acted as a disruptive force that catalyzed significant changes in both the content and delivery of health professions education, particularly in disciplines that rely heavily on hands-on clinical training.

The review findings emphasize the complementary nature of online and face-to-face instruction. Online learning has demonstrated effectiveness in delivering theoretical content and broadening access to education, especially under conditions that limit physical presence. However, in-person instruction remains essential for developing psychomotor skills, clinical reasoning, and interpersonal competencies-core elements of PT and other clinical education programs. These insights reinforce the importance of integrated hybrid models that combine the advantages of digital flexibility with the experiential depth of in-person learning.

As the global education landscape continues to evolve, sustained and systematic evaluation of digital clinical education models will be necessary to ensure their effectiveness in preparing competent healthcare professionals. Based on the synthesis of current evidence, several strategic directions can be identified to guide the future of PT education. First, hybrid education models should be standardized and optimized to ensure scalability and consistency across institutions. Second, instructional design should be aligned with advances in educational technologies and grounded in pedagogical theory. Third, efforts must be made to ensure equitable access to digital learning tools and infrastructure, particularly in underserved settings. Fourth, institutional support for faculty development and well-being should be strengthened to address the increased demands of digital teaching. Fifth, educational assessments should be reformed by adopting validated frameworks that accurately measure learning outcomes. Lastly, robust and adaptable educational infrastructures must be developed to ensure continuity and resilience in the face of future disruptions.

Collectively, these strategic recommendations aim to shape PT education into a more flexible, inclusive, and learner-centered system - one that is capable of addressing the evolving demands of healthcare and grounded in the principles of educational science.
